# The Effect of Repeated Electroacupuncture Analgesia on Neurotrophic and Cytokine Factors in Neuropathic Pain Rats

**DOI:** 10.1155/2016/8403064

**Published:** 2016-10-05

**Authors:** Junying Wang, Yonghui Gao, Shuping Chen, Chenlin Duanmu, Jianliang Zhang, Xiumei Feng, Yaxia Yan, Junling Liu, Gerhard Litscher

**Affiliations:** ^1^Institute of Acupuncture and Moxibustion, China Academy of Chinese Medical Sciences, Beijing 100700, China; ^2^Research Unit for Complementary and Integrative Laser Medicine, Research Unit of Biomedical Engineering in Anesthesia and Intensive Care Medicine and TCM Research Center Graz, Medical University of Graz, 8036 Graz, Austria

## Abstract

Chronic pain is a common disability influencing quality of life. Results of previous studies showed that acupuncture has a cumulative analgesic effect, but the relationship with spinal cytokines neurotrophic factors released by astrocytes remains unknown. The present study was designed to observe the effect of electroacupuncture (EA) treatment on spinal cytokines neurotrophic factors in chronic neuropathic pain rats. The chronic neuropathic pain was established by chronic constrictive injury (CCI). EA treatment was applied at Zusanli (ST36) and Yanglingquan (GB34) (both bilateral) once a day, for 30 min. IL-1*β* mRNA, TNF-*α* mRNA, and IL-1 mRNA were detected by quantitative real-time PCR, and the proteins of BDNF, NGF, and NT3/4 were detected by Western blot. The expression levels of cytokines such as IL-1*β* mRNA, TNF-*α* mRNA, IL-6 mRNA, and neurotrophic factors such as BDNF, NGF, and NT3/4 in the spinal cord were increased significantly after CCI. The astrocytes released more IL-1*β* and BDNF after CCI. Repeated EA treatment could suppress the elevated expression of IL-1*β* mRNA, TNF*α* mRNA, and BDNF, NGF, and NT3/4 but had no effect on IL-6 mRNA. It is suggested that cytokines and neurotrophic factors which may be closely associated with astrocytes participated in the process of EA relieving chronic pain.

## 1. Introduction

The pain information first activates the nociceptors in the periphery, which then project to the interneurons in the spinal cord. Then the pain information that goes through the ascending tracts activates the thalamic neurons and neurons of the cerebral cortex. The pain pathways that carry noxious information from peripheral nociceptors to higher levels of the central nervous system include a set of ascending pathways, as well as descending pathways that modulate that information. In the past, studies about pain focused on neurons; however, in recent years, the relationship between glial cells and pain has become a hot topic.

The chronic constriction injury (CCI) as a model of neuropathic pain induces hyperalgesia, allodynia, and spontaneous pain, which are expressions of neural plasticity. Previous research has indicated that CCI lowered the thermal and mechanical pain threshold [1, 2]. It has been increasingly recognized that spinal cord glial cells, including microglia and astrocytes, play a critical role in the induction and maintenance of neuropathic pain [3].

Astrocytes also release neurotrophic factors such as brain-derived neurotrophic factor (BDNF), glia cell line-derived neurotrophic factor (GDNF), nerve growth factor (NGF), and neurotrophin-3/4 (NT3/4). Their functions are not only to support the growth, survival, and differentiation of both developing and mature neurons in the central and peripheral nervous system, but also participating in the central and peripheral formation and development of neuropathic pain and regrowing damaged neurons. The expression of neurotrophic factors increased after nerve injuries [4]. The activated astrocytes also released different kinds of cytokine factors including tumor necrosis factor-alpha (TNF-*α*), interleukin-1 beta (IL-1*β*), interleukin IL-6, adenosine triphosphate (ATP), and substance P (SP) [5]. Those cytokine factors can activate pain-related neurons which release more factors, thus inducing pain in the spinal cord. The activated astrocytes together with the cytokine factors play a fundamental role in the process of chronic neuropathic pain [6]. The neurotrophic and cytokine factors are the key link between astrocytes and neurons in the generation of chronic pain.

The effect of electroacupuncture (EA) analgesia involves many different factors such as peripheral and central nervous-humoral regulation. EA uses the descending inhibitory pathway to make the brain send chemical substances down to the cells in the spinal cord to counteract the pain message sent up by the pain receptors [7]. Our previous study suggested that electroacupuncture (EA) has a cumulative analgesic effect on CCI rats, which is related with the cholinergic system and the extracellular signal-regulated kinases (ERK) signal pathway in the hippocampus and hypothalamus [8, 9]. EA intervention is effective in alleviating neuropathic pain in CCI rats, which may be closely related to its effects in lowering functional activity of NR 1 protein and mRNA in the spinal cord [10]. The central mechanism of EA-induced antihyperalgesia may be partially associated with a reduced expression of p-p38 MAPK (mitogen-activated protein kinase) and subsequently reducing the activation of OX-42 in neuropathic pain. Therefore, EA may be a new complementary and alternative therapy for neuropathic pain [11]. Several studies have indicated that EA has effects on neurotrophic factors such as BDNF and GDNF, suggesting that these factors are involved in the effectiveness of EA in the treatment of pain [12–14], but the relationship with astrocytes is not clear at the moment.

Based on the above research, we hypothesize that repeated EA treatment has some effects on the expression of neurotrophic and cytokine factors on astrocytes. Therefore, the purpose of this study is to investigate the effect of repeated EA on neurotrophic factors and cytokine factors in CCI rats.

## 2. Materials and Methods

### 2.1. Animals and Grouping

126 adult male Wistar rats (200–250 g), purchased from China Academy of Chinese Medical Sciences, were acclimatized to standard laboratory conditions (about 12 h alternate light-dark cycle) for a week and given free access to standard chow pellet diet and water. The rats were randomly assigned into different groups: normal group, sham CCI group, CCI group, sham+EA group (EA treatment for sham-CCI rats), and EA group (EA treatment for CCI rats). Normal animals and sham-operated rats were used as control.

The study was carried out in strict accordance with the recommendation in the Guidelines for Declaration of the National Institutes of Health Guide for Care and Use of Laboratory Animals (Publication number 80-23, revised 1996). All surgery was performed under anesthesia, and all efforts were made to minimize suffering.

### 2.2. Chronic Neuropathic Pain

The chronic pain model was established by ligature of the sciatic nerve with reference to modified Bennett's and Xie's methods [15]. Under anesthesia (using a mixture solution of ethyl urethane 0.28 mg/100 g plus chloralose (Sigma) 3.3 mg/100 g) and routine sterilization, the left sciatic nerve was exposed at the mid-thigh level by blunt dissection through the biceps femoris muscle. Four constrictive ligatures (4-0 surgical suture) were tied around the nerve at the distal end close to the bifid site at intervals of about 1.0 mm. The ligature was considered complete when a local mild muscular contraction of the leg could be clearly seen. After local application of an antibiotic (sodium penicillin, 9,000–10,000 U/rat), the muscle and skin were sutured in layers. For reducing experimental variability, all operations were finished by the same operator. In the sham-CCI group, the sciatic nerve was exposed but not tied.

### 2.3. Electroacupuncture Treatment

On the fifth day after operation (CCI or sham CCI), the acupoints Zusanli (ST36) and Yanglingquan (GB34) (both bilateral) were punctured with stainless-steel acupuncture needles of 0.20 mm in diameter (Gauge 28) to a depth of 4 mm, respectively, and stimulated electrically using Han's EA Stimulator (HANS-200A, made in China). EA treatment (2/100 Hz, 1 mA) was applied for 30 min daily, for 2 weeks.

### 2.4. Immunofluorescence

The rats were fixed by 4% paraformaldehyde through the circulatory system. The tissues of the spinal cord at L4-5 were taken out and put into 30% sucrose solutions overnight for dehydration. Then the tissues were sectioned and washed with the 0.1 M phosphate buffer (PB). The sections were blocked with 5% normal donkey serum in 0.01 M phosphate buffered saline triton (PBST) (135 mM NaCl, 4.7 mM KCl, 10 mM Na_2_HPO_4_, 2 mM NaH_2_PO_4_, and 1% TritonX-100) for 30 min and incubated with 1 : 500 rabbit anti-BDNF (SC-546, Santa Cruz Biotechnology, Dallas, TX, USA), 1 : 500 rabbit anti-IL1*β* (Abcam, Cambridge, UK), and 1 : 1000 mouse anti-GFAP (Cell Signaling Technology, Danvers, MA, USA) antibody overnight at 4°C. After washing with PBST three times, the sections were incubated with 1 : 300 Alexa Fluor 594-conjugated donkey anti-rabbit antibodies/1 : 300 Alexa Fluor 488-conjugated donkey anti-mouse (Invitrogen, Thermo Fisher Scientific, Waltham, MA, USA) at room temperature for 2 h. In the end, the sections were mounted on glass and covered with glycerinum. Fluorescence pictures were captured using a Nikon microscope (Nikon, Tokyo, Japan). For the negative control, the cells were stained without primary antibodies and showed no signals.

### 2.5. RNA Isolation and Quantitative Real-Time PCR (Polymerase Chain Reaction)

Total RNA was isolated from the spinal cord with Trizol (CW0581, CWbio, Beijing, China) and reverse transcribed to cDNA by PrimeScript*™* RT Reagent Kit with gDNA Eraser (Takara Bio, Shiga, Japan). The primer sequences used were as follows: IL-1*β*: forward 5′-CCCAACTGGTACATCAGCACCTCTC-3′, reverse 5′-CTATGTCCCGACCATTGCTG-3′; IL-6: forward 5′-GATTGTATGAACAGCGATGATGC-3′, reverse 5′-AGAAACGGAACTCCAGAAGACC-3′; TNF*α*: forward 5′-GGGCAGGTCTACTTTGGAGTCATTG-3′, reverse 5′-GGGCTCTGAGGAGTAGACGATAAAG-3′; GAPDH: forward: 5′-TGGAGTCTACTGGCGTCTT-3′, reverse 5′-TGTCATATTTCTCGTGGTTCA-3′. The reverse-transcribed products were amplified. Quantitative real time (QRT) PCR was performed in 96-well plates using the QRT PCR detection systems (AB7500, Applied Biosystems, Thermo Fisher Scientific, Waltham, MA, USA). All the cDNA samples were amplified in triplicate from the same RNA preparation and the mean value was calculated. Each reaction included 2 *μ*L of cDNA, 10 *μ*L of REALSYBRMixture (2x), 0.8 *μ*L (10 *μ*mol/*μ*L) of both forward and reverse primers, and 7.2 *μ*L of PCR-grade water, equating to a final volume of 20 *μ*L. PCR was performed under the following conditions: 10 min at 95°C, followed by 40 cycles of 15 s at 95°C, and 60 s at 60°C. After each cycle, fluorescence acquisition was performed. Finally, a dissociation curve was generated by increasing temperature from 65°C to 95°C in order to verify primer specificity. All samples for each reference gene were run on the same plate to avoid between-run variations. The relative expression was calculated in accordance with the ΔΔCt method. Relative mRNA levels were expressed as 2-ΔΔCt values.

### 2.6. Western Blot

The tissue of the spinal cord at L4-5 was excised. Total protein was extracted from the tissue by protein lysate containing protease and phosphatase inhibitors (Roche, Shanghai, China) using a tissue homogenizer. The protein concentration was determined with bicinchoninic acid (BCA) protein assay. An equal amount of protein in each sample was run on 5% or 8% sodium dodecyl sulfate polyacrylamide gel electrophoresis (SDS-PAGE) for about 60 min at 90/160 V and then electrotransferred onto polyvinylidene difluoride (PVDF) membrane (Millipore Corporation, Billerica, MA, USA) for 150 min at 90 mA. The membranes were blocked in 5% bovine serum albumin (BSA, Amresco, Solon, OH, USA) solution for 60 min at room temperature. The membranes were incubated with primary antibody BDNF protein (1 : 1000; SC-546, Santa Cruz Biotechnology, Dallas, TX, USA), NGF protein (1 : 2000; 1895-1, Epitomics, Burlingame, CA, USA), NT3 (1 : 10000; ab65804, Abcam, Cambridge, UK), NT4 (1 : 5000; sc-365444, Santa Cruz Biotechnology, Dallas, TX, USA), and GAPDH (1 : 20000, TDY042, Tiandeyue Biological Technology Company, Beijing, China) at 4°C overnight. After washing 3 times, the membranes were incubated with secondary antibody (1 : 20000 diluted of goat anti-rabbit Immunoglobulin (Ig) G or 1 : 10000 diluted of goat anti-mouse IgG) conjugated to horseradish peroxidase (Jackson Immuno Research Laboratories, West Grove, PA, USA) for 1 h at room temperature. The membranes were developed using an enhanced chemoluminescence (ECL) detection system to transfer to film. For densitometric analyses, the blots were scanned and quantified using TotalLab Quant analysis software (TotalLab Limited, Newcastle upon Tyne, UK). GAPDH was used as a loading control.

### 2.7. Statistical Analysis

The data collected in the present study are presented as mean ± standard deviation (mean ± SD) and analyzed by one-way repeated measures ANOVA, followed by post hoc test for least significant difference (LSD) to determine differences between two groups. Statistical significance was defined as *P* < 0.05.

## 3. Results

**Figure 1:**
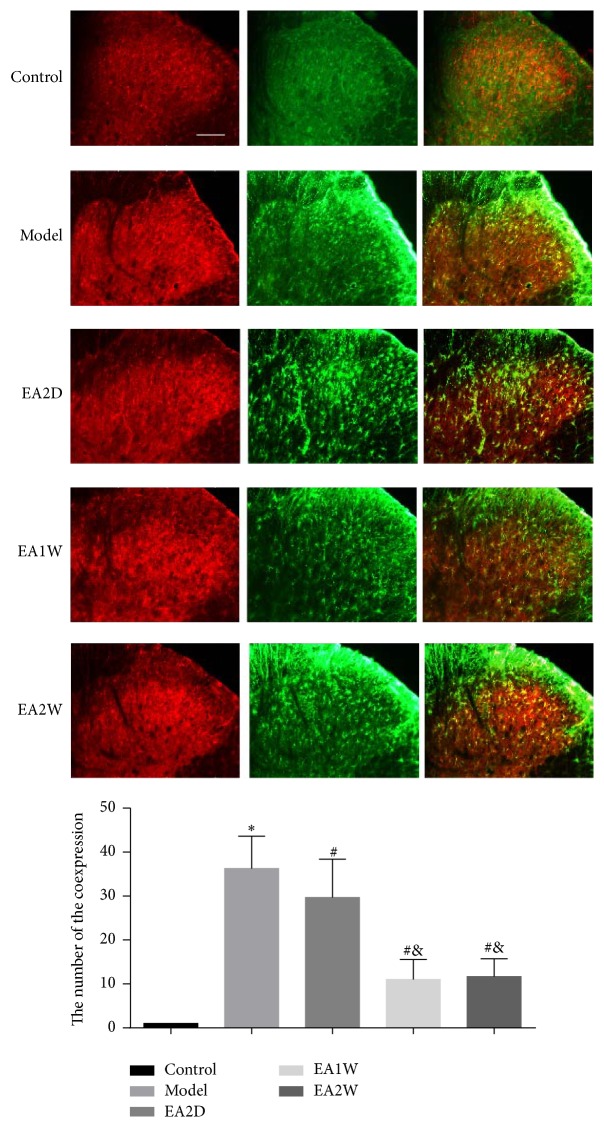
Effect of EA treatment on spinal IL-1*β* expression on astrocytes in different groups. The expression of IL-1*β* is shown in red and GFAP in green; the coexpression of IL-1*β* and GFAP is yellow. GFAP is the marker for the astrocytes, suggesting the expression of astrocytes (green). The histogram shows the count of the coexpression of IL-1*β* and GFAP (yellow). The yellow area represents the expression of IL-1*β* on the astrocytes. The coexpression level of IL-1*β* and GFAP (yellow) was increased after CCI and decreased after repeated EA treatment. Scale bar: 200 *μ*m. EA2D: after 2 days' EA treatment; EA1W: after 1 week's EA treatment; EA2W: after 2 weeks' EA treatment; ^**∗**^compared with the normal group; ^#^compared with the CCI group; ^&^compared with the CCI+EA2D group.

The main expression of IL-1*β* was on the III layer of the dorsal horn of the spinal cord. The expression of GFAP is the marker for the astrocytes. In the control group, the astrocytes were at rest, and there was almost no coexpression (yellow) with IL-1*β* (Figure 1). The astrocytes were activated after CCI. In the CCI model group, the cell body of astrocytes was relatively enlarged and the cell processes became thicker. There was more IL-1*β* expression and more IL-1*β* and GFAP coexpression (yellow) in the CCI model group. The coexpression was also mainly on the III layer of the dorsal horn of the spinal cord. It is suggested that chronic neuropathic pain activated the astrocytes to release more IL-1*β*. The coexpression of IL-1*β* and GFAP decreased after 1-week and 2-week EA treatment, but some IL-1*β* was still expressed on the astrocytes after EA treatment, even in the CCI+EA group after two weeks of treatment.

**Figure 2:**
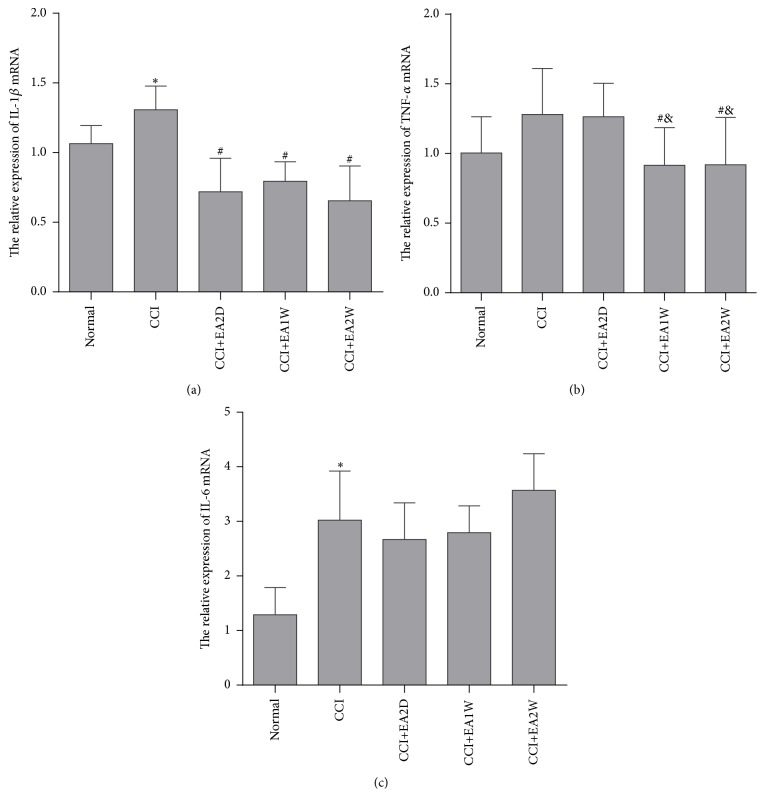
The relative expression of IL-1*β* mRNA, TNF-*α* mRNA, and IL-6 mRNA in the different groups (mean ± SD, *n* = 8). The expressions of IL-1*β* mRNA, TNF-*α* mRNA, and IL-6 mRNA were increased significantly after CCI and decreased after repeated EA treatment: ^**∗**^compared with the normal group; ^#^compared with the CCI group; ^&^compared with the CCI+EA2D group.

Compared with the control group, the relative expression of IL-1*β* mRNA increased significantly in the CCI group (*P* < 0.05; Figure 2). After EA treatment for 2 days, 1 week, and 2 weeks, respectively, the expression of IL-1*β* mRNA was decreased markedly (*P* < 0.05), suggesting that EA has an effect on IL-1*β* mRNA.

Compared with the control group, the relative expression of TNF-*α* mRNA increased significantly in the CCI group (*P* < 0.05; Figure 2). The relative expression of TNF-*α* mRNA in the CCI+EA2D group was little lower than the CCI group, but there was no significant difference (*P* > 0.05). Compared with the control group, the relative expression of TNF-*α* mRNA decreased markedly in the CCI+EA1W and CCI+EA2W group (*P* < 0.05).

Compared with the control group, the relative expression of IL-6 mRNA increased significantly in the CCI group (*P* < 0.05). After EA treatment for 2 days, 1 week, or 2 weeks, the expression of IL-6 mRNA showed no significant change (*P* > 0.05) (Figure 2).

It is suggested that EA analgesia is connected with the downregulated expression of IL-1*β* mRNA and TNF-*α* mRNA but has no effect on IL-6 mRNA expression.

**Figure 3:**
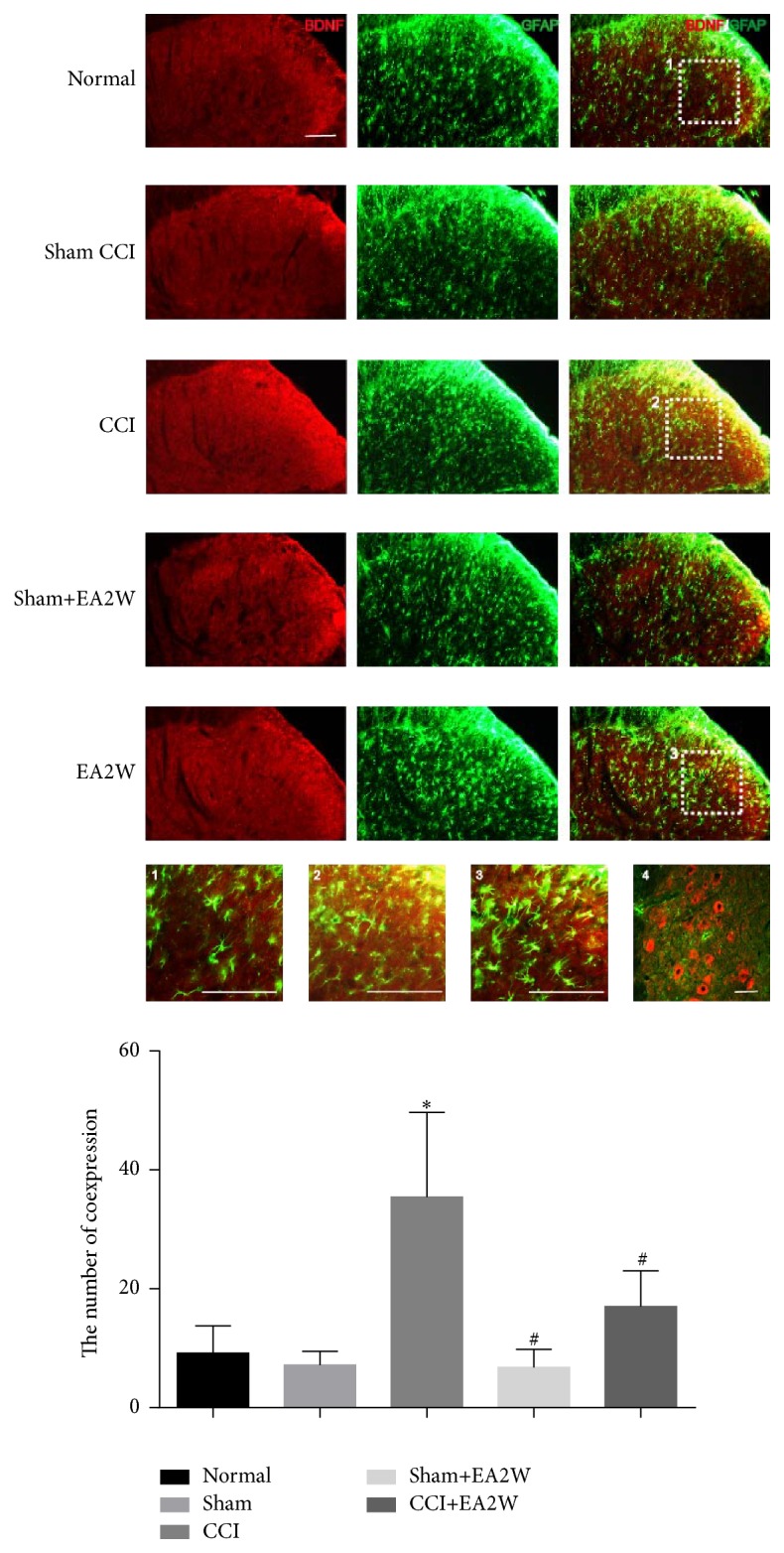
Effect of EA treatment on spinal BDNF expression on astrocyte in different groups. The expression of BDNF is shown in red and that of GFAP in green; the coexpression of BDNF and GFAP is yellow. The histogram shows the cell number of the coexpression of BDNF and GFAP (yellow). The yellow area represents the expression of BDNF on the astrocytes. The coexpression level of BDNF and GFAP (yellow) was increased after CCI and decreased after 2 weeks' EA treatment (EA2W). Pictures 1, 2, and 3 represent magnifications of the merged image in the normal group, the CCI group, and EA2W group, respectively. Picture 4 shows the merged image in the spinal anterior horn after CCI. Scale bar: 200 *μ*m; ^**∗**^compared with the normal group; ^#^compared with the CCI group.

BDNF was mainly expressed on layers I and II and some on layer III of the dorsal horn of the spinal cord in the normal group (Figure 3). There was almost no coexpression of BDNF and GFAP in the normal group. No significant change was detected including the state of astrocytes and the coexpression level of BDNF and GFAP (yellow) after the sham operation, suggesting that the sham operation had little effect on the astrocytes and the expression of BDNF. After giving EA treatment for 2 weeks on the sham rats, there was still no overexpression of BDNF and GFAP. It is suggested that EA treatment had no effect on the sham rats. The astrocytes were activated: the cell body was relatively enlarged and the cell processes became thicker in CCI group. The expressions of BDNF on astrocytes were also markedly higher than in the normal group and the sham group (yellow, Figure 3, Picture 2). Compared with the CCI group, the coexpression of BDNF and GFAP was significantly decreased (more green and less yellow, Figure 3, Picture 3), suggesting that EA inhibited the expression of BDNF on astrocyte to exert its analgesic effect on the CCI rats. There was no coexpression of BDNF and GFAP in the spinal anterior horn in CCI group (Figure 3, Picture 4).

**Figure 4:**
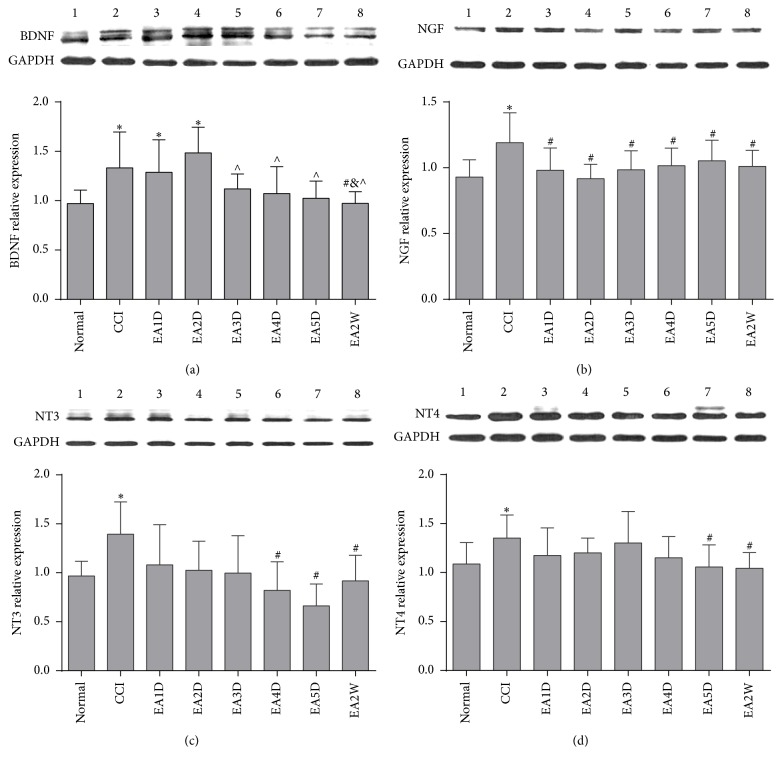
The relative expression of BDNF, NGF, and NT3/4 in different groups (mean ± SD, *n* = 7). The expression of BDNF, NGF, and NT3/4 was increased significantly after CCI. The expression of BDNF and NT3/4 was decreased after repeated EA treatment, and the expression of NGF was decreased immediately after only one EA treatment: ^**∗**^compared with the normal group; ^#^compared with the CCI group; ^&^compared with the CCI+EA1D group; ^∧^compared with the CCI+EA2D group.

After CCI operation, the expression of BDNF in the spinal cord was higher than in the normal group (*P* < 0.05, Figure 4(a)), suggesting that BDNF is involved in the process of chronic pain. Compared with the normal group, the expressions of BDNF in the CCI+EA1D and CCI+EA2D group were still upregulated markedly (*P* < 0.05). Compared with the CCI group, the expression of BDNF at EA2W was downregulated significantly (*P* < 0.05), almost approaching the control level. The expression of NGF in the CCI group was higher than the expression of the normal group (*P* < 0.05, Figure 4(b)). After only one EA treatment, the expression of NGF decreased (*P* < 0.05). Compared with the normal group, the expression of NT3/4 increased observably (*P* < 0.05) in the CCI group. Until the fourth day of EA treatment, the expression of NT3 decreased markedly (*P* < 0.05), and the expression of NT4 also decreased, but not significantly (*P* > 0.05). Compared with the CCI group, the expression of NT4 decreased markedly (*P* < 0.05) in EA 5D and EA 2W group. EA treatment had a cumulative effect on the expression of NT3/4. It is suggested that EA analgesia is connected with the downregulated expression of BDNF, NT3/4, and NGF.

## 4. Discussion

Chronic pain is pain that lasts a long time. Other health problems, such as fatigue, sleep disturbance, decreased appetite, and mood changes, often accompany chronic pain. Therefore, chronic pain severely affects the quality of life of the sufferers [16]. Because of the serious side effects of drug treatment and the high cost, the treatment of chronic pain remains one of the most challenging problems and was studied by many research groups. Chronic pain moves along the nerve fibers of the body to the dorsal horn of the spinal cord; then it goes up to the brain and separates and terminates in the hypothalamus and the limbic structures. The spinal cord plays an important part in the ascending pain pathway from the body to the brain.

On the spinal cord, the astrocytes were activated by cytokines and neurotrophins which are secreted by microglia or neurons, and then the activated astrocytes released more cytokines and neurotrophins which caused the pain signal cascade to enlarge [17, 18]. The cytokines such as IL-1*β*, IL-6, and TNF-*α* and neurotrophins such as BDNF, NT3/4, and NGF play an important role on the spinal cord under neuropathic pain [19, 20].

IL-1*β* mRNA was upregulated in the injured sciatic nerve (SCN) after partial sciatic nerve ligation (PSL), and PSL-induced neuropathic pain was prevented by the perineural injection of anti-IL-1*β* in mice [21]. The important mediator in neuropathic pain, TNF-*α*, increased already after 6 h in the SCN, and 1 day after surgery in dorsal root ganglia (DRG) [22]. Ligation of L5-L6 spinal nerves (SNL) resulted in an upregulation of IL-6 mRNA expression in dorsal root ganglia and IL-6 concentration in the dorsal spinal cord [23]. Intrathecal (i.t.) administration of IL-1*β* and endoneural injection of TNF-*α* or IL-6 induced allodynia and hyperalgesia in rats [19, 24, 25]. TNF-*α* and IL-1*β* mRNA levels increased immediately after spinal nerve transection and remained high for 6 d, while IL-6 transcripts only began to increase after 12 h [26]. The cytokine cascades are involved in the development of neuropathic pain and may lead to the development of new and more efficient medications for many types of pain [27].

In our experiment, the expressions of IL-1*β* mRNA, TNF-*α* mRNA, and IL-6 mRNA were still increased in the spinal cord 18 days after CCI. The nerve injury activated the neurons and microglia and released the cytokines which activated the astrocytes [18]. The activated astrocytes also released the cytokines including IL-1*β*, so the coexpression of GFAP and IL-1*β* was increased.

It is well known that neurotrophic factors play a crucial role in regulating the survival and specification of the nervous system, but it is now well established that neurotrophic factors have an important influence on the generation of pain [28]. Overexpression of BDNF in the dorsal horn and mechanical allodynia induced by SNL might activate astrocytes in neuropathic pain in rats [29]. The presence of NT3 was increased mainly in the small DRG cells of neuropathic animals [30]. NGF acts as an important intermediate in inflammatory pain, contributing to both peripheral and central sensitization [31]. NGF expression increased in the dorsal root ganglion during inflammation, and anti-NGF could be an alternative drug for painful conditions [32, 33]. The increased expression of IL-1*β* and SP and impaired GDNF production were associated with the decreased mechanical responses 3 days after surgery (trigeminal neuropathic pain) [34]. In normal mice, i.t. administration of BDNF produces an acute, dose-dependent thermal hyperalgesic response [35], and intradermal injection of NGF into the plantar skin of adult rat hindpaws produced a prolonged and stable thermal hyperalgesia to radiant heat [35].

In our research, we found that CCI induced the overexpression of BDNF, NGF, and NT3/4. BDNF expression was upregulated in small and medium-sized DRG after inflammation and nerve injury [36, 37]. It was also upregulated in the astrocyte in our experiment after chronic constriction of the SCN. The coexpression of BDNF and GFAP was increased after CCI, suggesting the astrocyte released more BDNF after CCI which contributed to chronic neuropathic pain.

Du proposed that neurotrophins and cytokines play a role in the mechanism of therapeutic effect of acupuncture [38]. So far, however, there is no systematical study on the effect of acupuncture on the proinflammatory cytokine and neurotrophic factor expression. Previous research proved that EA has superior effects on proinflammatory cytokines compared to simple acupuncture in patients with rheumatoid arthritis [39], which is why in our experiment we observed the effect of repeated EA treatment on proinflammatory cytokines and neurotrophic factors in chronic pain rats.

Experiments in different model animals showed that EA treatment could noticeably decrease the elevated expression levels of proinflammatory cytokines including IL-1*β*, TNF-*α*, and IL-6 in inflamed skin tissues [40], serum [41], peripheral nerves, and DRG [42], even synovia [43], after injection of complete Freund's adjuvant, nerve injury, knee osteoarthritis, or spinal cord ischemia-reperfusion injury [44]. In our experiment, we demonstrated that repeated EA treatment suppresses the upregulated expression levels of IL-1*β* and TNF-*α* mRNA after CCI injury in the spinal cord but had no significant effect on the expression levels of IL-6 mRNA. This suggests that EA treatment could suppress the elevated levels of spinal proinflammatory cytokines after nerve injury, which may contribute to the effect of EA in treatment for chronic neuropathic pain. The expression of IL-1*β* on astrocytes decreased after EA treatment. Sun reported that EA can effectively suppress CCI-induced upregulation of expression of spinal GFAP, TNF-*α* mRNA, and IL-1*β* mRNA [45], which may be connected with reducing mechanical allodynia and thermal hyperalgesia in neuropathic pain rats.

As mentioned above, one of the important functions for neurotrophic factors is survival and specification of the nervous system, so the mechanism of EA on neurotrophic factors is similar but not identical to the effect of EA on cytokine factors after nerve injury. A previous study suggested that EA decreased the injury of the spinal cord and increased the BDNF mRNA expression to induce the regeneration and repair of nerves [46]. The expression of BDNF and NGF also increased significantly after 3 days of EA [47]. In our experiment, the expression of BDNF in the spinal cord was most increased after 2 days of EA to repair the injured nerve and then started to be downregulated after 3 days of EA, which may be a result of less proinflammatory cytokine release after repeated EA treatment. EA treatment has a cumulative effect on expression of BDNF after 4 days of treatment. The downregulated expression of BDNF could be noticed on the astrocytes after 2 weeks' EA treatment. The repeated EA also increased the expression of NT3/4 but had no effect on the expression of NGF. The expression of NGF was decreased significantly after the first EA treatment, which may be because NGF is closely related with pain and is sensitive to EA treatment. A previous study about the emerging links between EA and NGF investigated the role of NGF as a mediator of EA effects in the central nervous system and as a modulator of sensory and autonomic activity [48]. Low-frequency EA could reduce side effects like hypersensitivity and hyperalgesia induced by NGF administration [49]. The efficacy of EA might depend on its actions on spinal/peripheral NGF synthesis/utilisation and normalization [50].

In summary, it can be stated that EA treatment is closely connected with IL-1*β* and TNF-*α* mRNA and BDNF, NGF, and NT3/4, but not with IL-6. EA suppressed the elevated expression of cytokine and neurotrophic factors released by astrocytes, which may be one of the mechanisms of repeated EA analgesia in chronic pain rats.
